# Clonal status and clinicopathological observation of cervical minimal deviation adenocarcinoma

**DOI:** 10.1186/1746-1596-5-25

**Published:** 2010-04-24

**Authors:** Li Gong, Wen-Dong Zhang, Xiao-Yan Liu, Xiu-Juan Han, Li Yao, Shao-Jun Zhu, Miao Lan, Yan-Hong Li, Wei Zhang

**Affiliations:** 1 Department of Pathology, Tangdu Hospital, the Fourth Military Medical University, Shaanxi Xi'an 710038, China; 2 Department of Nuclear Medicine, Tangdu Hospital, the Fourth Military Medical University, Shaanxi Xi'an 710038, China

## Abstract

**Background:**

Minimal deviation adenocarcinoma (MDA) of the uterine cervix is defined as an extremely well differentiated variant of cervical adenocarcinoma, with well-formed glands that resemble benign glands but show distinct nuclear anaplasia or evidence of stromal invasion. Thus, MDA is difficult to differentiate from other cervical hyperplastic lesions. Monoclonality is a major characteristic of most tumors, whereas normal tissue and reactive hyperplasia are polyclonal.

**Methods:**

The clinicopathological features and clonality of MDA were investigated using laser microdissection and a clonality assay based on the polymorphism of androgen receptor (AR) and X-chromosomal inactivation mosaicism in female somatic tissues.

**Results:**

The results demonstrated that the glands were positive for CEA, Ki-67, and p53 and negative for estrogen receptor (ER), progesterone receptor (PR), and high-risk human papilloma virus (HPV) DNA. The index of proliferation for Ki-67 was more than 50%. However, the stromal cells were positive for ER, PR, vimentin, and SM-actin. The clonal assay showed that MDA was monoclonal. Thus, our findings indicate that MDA is a true neoplasm but is not associated with high-risk HPV.

**Conclusions:**

Diagnosis of MDA depends mainly on its clinical manifestations, the pathological feature that MDA glands are located deeper than the lower level of normal endocervical glands, and immunostaining.

## Background

Minimal deviation adenocarcinoma (MDA) of the uterine cervix, also known as adenoma malignum, is defined as an extremely well-differentiated variant of cervical adenocarcinoma. One percent to 3% of all cervical glandular malignancies fit this definition [1]. Distorted glands are the main component of these malignancies, while the stromal spindle cells are responsive hyperplasia. The diagnosis of MDA is based on histopathological characteristics--the presence of well-formed glands and stromal invasion--and immunophenotype: CEA positive reactivity and a high proliferative index of Ki-67. However, MDA is difficult to differentiate from benign hyperplastic lesions such as microglandular hyperplasia, adenomatous hyperplasia, diffuse laminar endocervical glandular hyperplasia, and lobular endocervical glandular hyperplasia. Monoclonality is a major characteristic of most tumors. By contrast, normal tissue and reactive hyperplasia are polyclonal [2]. The clonality of MDA has not been reported in the literature. Therefore, we sought to elucidate its clonality using laser microdissection and a clonality assay based on the polymorphism of androgen receptor (AR) and X-chromosomal inactivation mosaicism in female somatic tissues. At the same time, we investigated MDA's clincopathological features and immunohistochemical characteristics.

## Materials and methods

### Samples

All seven cases of samples, including 3 cases of MDA, 1 adenomatous hyperplasia, and 3 hysteromyoma (controls), were collected between January 2008 and September 2009 from Tangdu Hospital, the Fourth Military Medical University (Xi'an, China). The study protocol was approved by the Medical Ethics Commission of the Fourth Military Medical University in Xi'an. All samples were surgically resected, fixed in 4% formaldehyde, and embedded in paraffin. Serial sections were cut and stained with hematoxylin and eosin (HE). Each case was examined by three pathologists and diagnosed according to the morphological criteria for MDA, adenomatous hyperplasia, and hysteromyoma.

### Immunohistochemistry

Immunostaining was carried out using a streptavidin-labeled peroxidase (S-P) kit (KIT9730) according to the manufacturer's instructions. The primary antibodies used in this study included mouse anti-human monoclonal antibodies (mAb) against carcinoembryonic antigen (CEA), cytokeratin (CK)18, cancer antigen (CA)125, estrogen receptor (ER), desmin, Ki-67, p53, and progesterone receptor (PR); rabbit anti-human polyclonal antibodies against S-100 protein and smooth muscle actin (SM-actin); and mouse anti-pig mAb against vimentin. All of the reagents for immunostaining were supplied by Maixin Biotechnology Corporation Limited, Fuzhou, China.

### In situ hybridization

In situ hybridization for high-risk human papillomavirus (HPV) was performed using a commercially available biotinylated HPV DNA probe mixture for HPV types 16 and 18 (Panpath, Holland) according to the manufacturer's instructions. Briefly, a 4-μm-thick formalin-fixed, paraffin-embedded sample tissue section was deparaffinaged in xylene and pretreated by microwave irradiation and pepsin digestion at 37°C for 30 minutes. Approximately 4 μL of the HPV DNA probe mixture was dropped on a pretreated sample tissue section, denatured at 95°C for 5 minutes, and hybridized at 37°C for 16 hours using a programmable temperature control system (ThermoBrite, USA). The slide was washed, incubated with streptavidin-alkaline phosphatase solution (Maixin), and then visualized with nitroblue tetrazolium/bromo-4-chloro-3-indolyphosphatase. For the evaluation, samples were considered positive for high-risk HPV when >10% of glandular or cancerous cell nuclei were stained positively.

### Microdissection and DNA extraction

**Figure 1:**
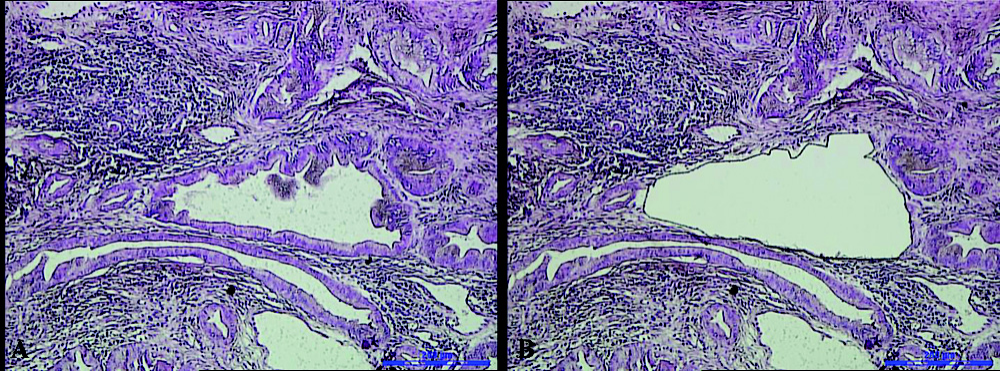
**A distorted gland before laser microdissection (1A); after laser microdissection (1B)**.

Eight 10-μm tissue sections (1.0 × 1.0 cm^2^), obtained from representative paraffin blocks, were placed on a UV-absorbing membrane and underwent laser microdissection by LMD6000 (Leica Microsystems Ltd., Wetzlar, Germany). After HE-staining, the slides were mounted on a microstat, and the distorted glands were then dissected by UV laser using the motorized optical-beam scanning mode (Figure 1). The dissected (with the attached specimen) was dropped by gravity into the cap of 0.5-mL microcentrifuge tube filled with 40 μL lysate buffer and 10 μL proteinase K. For each dissected lesion, stromal cells of approximately the same area were isolated and analyzed as a control. The microcentrifuge tubes were then placed in a water bath (48°C) to digest the tissues. After digestion for 12 to 20 h, genomic DNA was extracted and examined by 2% agarose gel electrophoresis, then stored at -20°C.

### Polymerase chain reaction (PCR) amplification for clonal assay

Nested PCR was used to amplify detection of the length polymorphism of CAG short-tandem repeat (STR) in exon 1 of the AR gene loci as described previously [3]. The genomic DNA extracted from lesions and from from non-lesion tissues (for the AR gene, 10 μL each) was incubated with 5 U of *Hha *I (Promega, Madison, WI, USA) at 37°C for 3 h in a volume of 20 μL containing 2 μL of 10× reaction buffer. The digested DNA samples, 1 μL each, were then subjected to nested PCR. The reaction mixture was 50 μL in volume, containing 4 μL of 10 mM dNTP (Gibco BRL, Life Technologies, Inc., Gaithersburg, MD, USA), primers AR1A and AR1B (20 μM each), and 5 μL of 10× buffer, 1.5 μL of 50 mM MgCl_2_, and 2.5 U of Taq DNA polymerase (Gibco BRL). The amplification was conducted using a PT-200 thermocycler (MJ Research, Inc., Watertown, MA, USA) for 25 cycles (94°C for 40 sec, 56°C for 50 sec, and 72°C for 1 min). For the second round, PCR products (1 μL) were used as templates for a second PCR reaction using primers AR2A and AR2B. The amplification procedure was the same as that in the first round. Finally, the amplification efficiency was checked by resolving PCR reaction aliquots on 2% agarose gels. The PCR products, 4 μL for each, were also mixed with the same volume of loading buffer (1 g/L xylene cyanole, 1 g/L bromophenol blue, in formamide) and resolved on an 8% polyacrylamide gel containing 8 mol/L urea using the Mini-VE system (Amersham Biosciences, San Francisco, CA, USA) at 120 V for 4 h. Bands were visualized using silver staining.

### Analysis and assessment of PCR products for clonal assay

Images of PCR gels were recorded, and the intensities of the PCR bands for both alleles were quantitated using an image-analyzing system (LabWorks 3.0, UVP, Cambridge, UK). A reduction in fluorescence intensity of at least 50% for either band, as compared with the intensity of the bands obtained in the absence of *Hpa *I digestion, was used as indicator of loss of X chromosome inactivation mosaicism [3]. A corrected ratio (CR) was calculated by the ratio of the upper-band intensity to the lower-band intensity, or vice-versa, of the same sample before and after digestion to give a CR value >1. In the present study, a CR value ≥ 2 was used to define loss of X chromosome inactivation mosaicism.

## Results

### Pathological observation

**Figure 2:**
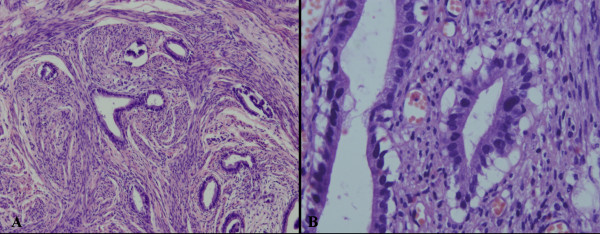
**Histopathological characteristics of MDA**. Microscopically, the lesion was characterized by mucinous glands which resembled normal endocervical glands, but showing distinct nuclear anaplasia or evidence of stromal invasion (A). Mitotic figures might be found (B). (A, Magnification × 100; B, Magnification × 400)

Microscopically, lesions were characterized by mucinous glands that resembled normal endocervical glands but showed distinct nuclear anaplasia or evidence of stromal invasion. Mitotic figures was found (Figure 2).

### Immunohistochemical analysis

**Figure 3:**
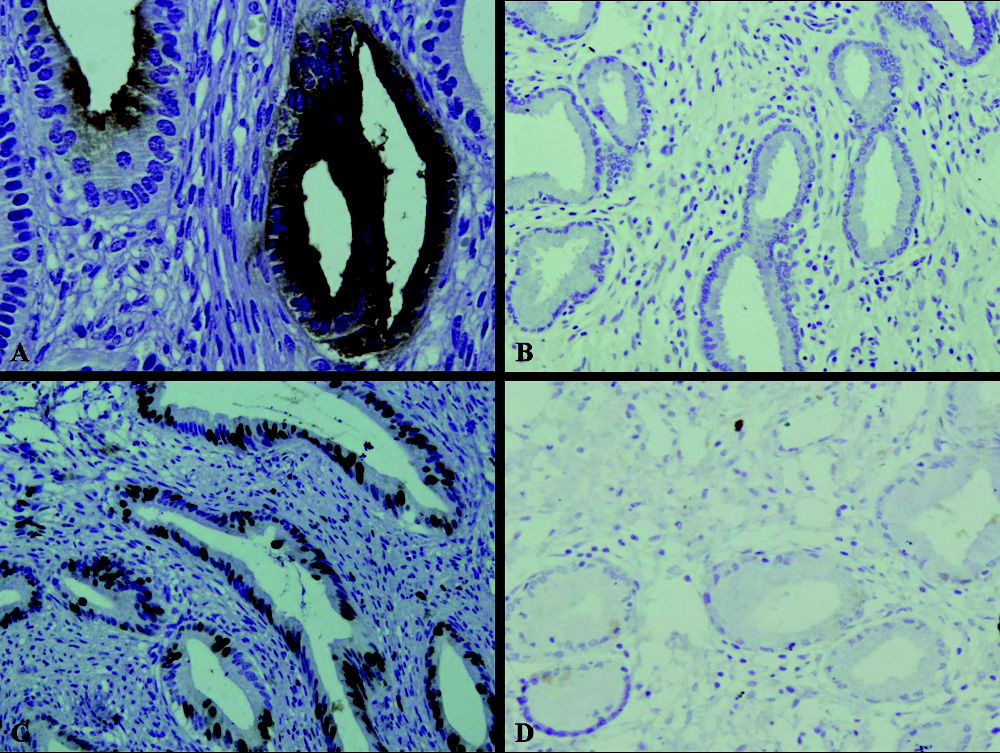
**The immunohistochemical staining of CEA and Ki-67**. The expression of CEA and Ki-67 were found in the glandular epithelium of MDA (A, C), respectively, but no in the normal cervical tissues (B, D).

**Figure 4:**
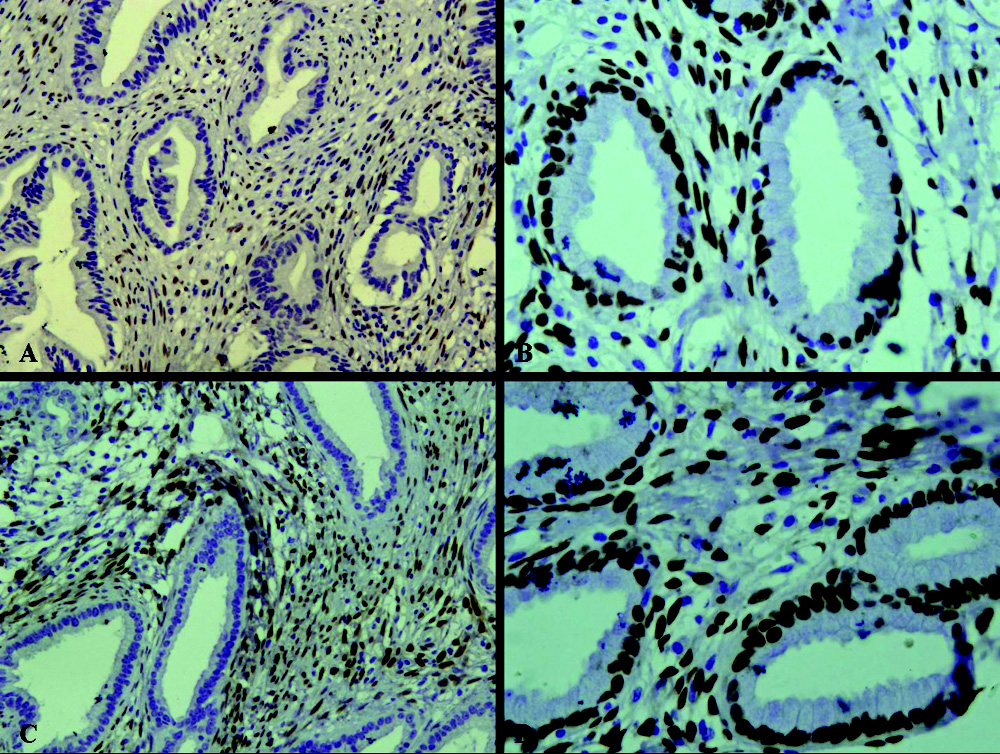
**The immunohistochemical staining of ER and PR**. ER and PR were found to be positive in the normal cervical tissues and stromal cells (B, D), but negative in the glandular epithelium and positive in the stromal cells of MDA (A, C).

**Figure 5:**
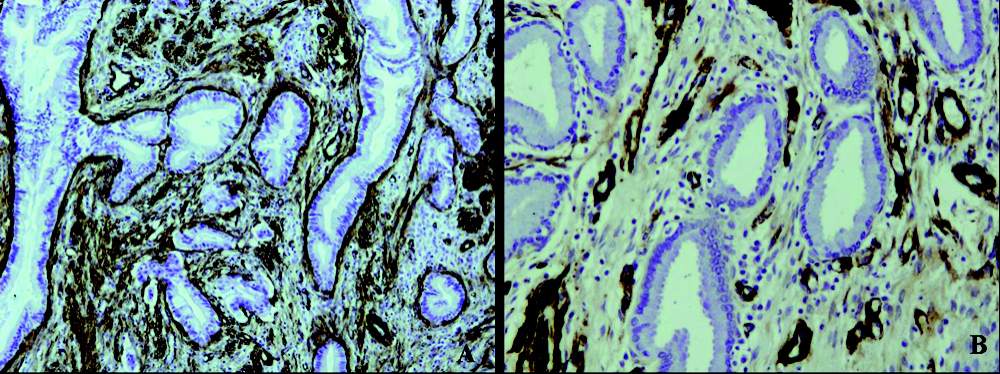
**The immunohistochemical staining of SM-actin**. The stromal cells were also positive for SM-actin in both MDA and the normal cervical tissues, but the stromal cells with SM-actin positive reactivity were more abundant in MDA (A) than in the normal cervical tissues (B).

It showed that CEA, Ki-67 and p53 were expressed in the glandular epithelium of MDA, but not in the normal cervical tissues (Figure 3). Moreover, the normal cervical tissues and stromal cells were found to be positive for ER and PR, but the glandular epithelium were negative for these receptors (Figure 4). Stromal cells were also positive for SM-actin and vimentin in both MDA and the normal cervical tissues, but stromal cells with SM-actin positive reactivity were more abundant in MDA than in the normal cervical tissues (Figure 5).

### In situ hybridization

As for in situ hybridization, all 3 MDAs examined were negative for high-risk HPV DNA, but blue grandular nuclei were found in the cervical tissue with positive control for HPV types 16 or 18.

### Clonality determination

**Figure 6:**
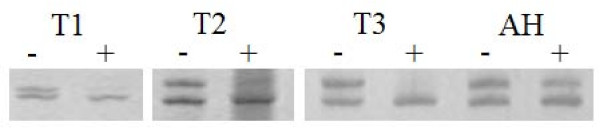
**Electropherogram on 8% polyacrylamide gel**. On the AR PCR gel pictures, there was two bands before DNA samples obtained from glandular epithelium was digested by *Hha *I. In contrast, one band disappeared after the DNA samples of the glandular epithelium was digested by *Hha *I. However, the intensities of the two bands were equal for a case of adenomatous hyperplasia and three cases of normal cervical tissues with or without *Hha *I digestion -, pretreated without *Hha *I; +, pretreated with *Hha *I; M, DNA marker. T1-T3, MDA tissues from 3 different female patients. AH, adenomatous hyperplasia.

The clonality assay demonstrated polymorphism for all the samples at AR loci. Before digestion by *Hha *I, PCR gels of DNA samples obtained from glandular epithelium showed two AR bands. After *Hha *I digestion, one band was no longer evident. However, regardless of *Hha *I digestion, the intensities of the two bands were equal for all the stromal cells, the case of adenomatous hyperplasia, and the 3 cases of normal cervical tissues (Figure 6). This result suggests that MDA can be interpreted as a neoplastic hyperplasia.

## Discussion

Minimal deviation adenocarcinoma (MDA) of the uterine cervix, also known as adenoma malignum, is a highly differentiated form of adenocarcinoma, representing about 1% to 3% of all endocervical adenocarcinomas [1]. It was first described as adenoma malignum by Gusserow in 1870 [4]. The term "minimal deviation adenocarcinoma" for extremely well-differentiated adenocarcinoma of the uterine cervix was proposed by Silverberg and Hurt in 1975 [5].

The average age of patients described in the literature ranges from 42.6 to 53.3 years, and the youngest patient reported was 16 years [6,7]. The average age of our three patients was 39 years. The most common presenting symptoms (in decreasing order of frequency) were watery discharge, menometrorrhagia, irregular genital bleeding, and abdominal swelling.

Human papillomavirus (HPV) is widely considered to be related to cervical carcinogenesis, and it is always detected in squamous cell carcinoma and its precursors. The oncoproteins E6 and E7, which are encoded in high-risk HPV types 16 and 18, typically are found in these lesions. A high incidence of HPV also has been reported in adenocarcinoma and adenocarcinoma in situ of the uterine cervix [8,9]. However, recent studies that have reported a rather low incidence of HPV in MDAs suggest that MDA is probably not related to HPV infection [10,11]. In this study, we investigated HPV infection in MDA using in-situ hybridization technique, but we did not find glandular nuclei that were positive for high-risk HPV, a negative finding compatible with those of previous studies. McGowan et al. [12] have reported that Peutz-Jeghers syndrome, a rare hereditary autosomal disorder characterized by benign hamartomatous polyposis in the gastrointestinal tract and mucocutaneous pigmentation, accompanied by ovarian mucinous tumors, may sometimes be complicated by MDA. However, our patients included no cases of Peutz-Jeghers syndrome.

The diagnosis of MDA is based on histopathological characteristics and immunophenotype. Microscopically, the glands are irregular in size and shape and lined predominantly by mucin-containing columnar epithelial cells with basal nuclei. Moreover, they typically exhibit deep invasion of the cervical wall. Immunohistochemically, glands are positive for CEA and Ki-67 but negative for ER, PR, and CA125. In our study, the results of histopathological and immunohistochemical observation were compatible with this description. In addition, we found that ER and PR were expressed in the stromal cells of both MDA and normal cervical tissues, a finding not described in the literature. However, it is almost impossible to histologically differentiate these tumor glands from normal endocervical glands, because the tumor glands of MDA are highly differentiated. Monoclonality is one of the main features of most tumors, whereas normal and reactive hyperplastic lesions are polyclonal [2]. The clonality assay we used is based on X-chromosome inactivation mosaicism and polymorphism at the AR loci in female somatic cells. Each female somatic cell contains two X chromosomes, one of which is inactivated randomly by methylation during early embryogenesis, while the other preserves its genetic activity throughout life. AR polymorphism shows different lengths of the CAG short-tandem repeat (STR) at exon 1 [13]. After digestion with the methylation-sensitive restriction enzymes *Hha *I, the normal and reactive hyperplastic tissues with polymorphism show two alleles with equal intensity. Neoplastic tissues show only one of the two alleles, with obviously reduced intensity [2].

In our study, the result of the clonal assay demonstrated that MDA was a neoplastic lesion, whereas adenomatous hyperplasia and normal cervical tissues were polyclonal, further confirming the nature of MDA. Understanding this distinction will help clinicians to differentiate MDA from benign hyperplastic lesions such as microglandular hyperplasia, adenomatous hyperplasia, diffuse laminar endocervical glandular hyperplasia, and lobular endocervical glandular hyperplasia.

PCR-based clonality analysis occasionally produces a pseudomonoclonal or pseudopolyclonal result [14]. The major mechanism responsible for the pseudomonoclonal result is patch size [15,16]. If progeny cells remain adjacent to each other after cell division, large patches of cells are formed, all of which contain an identical pattern of X-chromosome inactivation. Lesions arising from two, three, or more cells within a patch will show the same pattern of X-chromosome inactivation and will thus appear monoclonal. This concept is a particularly important consideration when using very small tissue samples obtained by microdissection. To avoid this problem, Diaz-Cano et al. [14] recommend using sample sizes larger than 100 cells or 0.25 cm^2^. Although we performed laser microdissection in our study to obtain the pure glands, the sample was composed of numerous different glands, and sample sizes were about 1000 cells. The reason for pseudopolyclonal results is likely contamination caused by a heavy inflammatory cell infiltrate or inadvertent inclusion of stromal cells. We used laser microdissection to avoid this circumstance. Therefore, we believe that our result is reliable. However, additional studies with larger sample sizes are necessary to conclusively prove our hypothesis.

In summary, MDA is a highly differentiated form of adenocarcinoma. The results of clonal assay indicate that MDA is a true neoplasm but is not associated with high-risk HPV. Diagnosis of MDA mainly depends on its clinical manifestations and the pathological feature that MDA glands are located deeper than the lower level of normal endocervical glands. Immunostaining is extremely helpful in diagnosing MDA.

## Abbreviations

MDA: minimal deviation adenocarcinoma; AR: androgen receptor; PCR: Polymerase chain reaction; CR: corrected ratio; HPV: human papillomavirus; CEA: carcinoembryonic antigen; CK: cytokeratin; CA (cancer antigen)125; ER: estrogen receptor; SM-actin: smooth muscle actin; PR: progesterone receptor

## Competing interests

The authors declare that they have no competing interests.

## Authors' contributions

LG selected the research topic, participated in the study, and wrote the manuscript. WDZ participated in writing the manuscript. YHL and WZ provided grant support. XYL and LY participated in the study. SJZ and XJH conducted the pathological examination. ML provided the technique support. All authors have read and approved the final manuscript.
